# An active self-cleaning surface system for photovoltaic modules using anisotropic ratchet conveyors and mechanical vibration

**DOI:** 10.1038/s41378-020-00197-z

**Published:** 2020-09-21

**Authors:** Di Sun, Karl F. Böhringer

**Affiliations:** 1 Department of Electrical and Computer Engineering, University of Washington, Seattle, WA 98195 USA; 2 Institute for Nano-Engineered Systems, University of Washington, Seattle, WA 98195 USA

**Keywords:** Electrical and electronic engineering, Environmental, health and safety issues

## Abstract

The purpose of this work is to develop an active self-cleaning system that removes contaminants from a solar module surface by means of an automatic, water-saving, and labor-free process. The output efficiency of a solar module can be degraded over time by dust accumulation on top of the cover glass, which is often referred to as “soiling”. This paper focuses on creating an active self-cleaning surface system using a combination of microsized features and mechanical vibration. The features, which are termed anisotropic ratchet conveyors (ARCs), consist of hydrophilic curved rungs on a hydrophobic background. Two different ARC systems have been designed and fabricated with self-assembled monolayer (SAM) silane and fluoropolymer thin film (Cytop). Fabrication processes were established to fabricate these two systems, including patterning Cytop without degrading the original Cytop hydrophobicity. Water droplet transport characteristics, including anisotropic driving force, droplet resonance mode, cleaning mechanisms, and system power consumption, were studied with the help of a high-speed camera and custom-made test benches. The droplet can be transported on the ARC surface at a speed of 27 mm/s and can clean a variety of dust particles, either water-soluble or insoluble. Optical transmission was measured to show that Cytop can improve transmittance by 2.5~3.5% across the entire visible wavelength range. Real-time demonstrations of droplet transport and surface cleaning were performed, in which the solar modules achieved a 23 percentage-point gain after cleaning.

## Self-cleaning solar power: Micro-patterned panels make water do the work

Micro-patterned, self-cleaning solar panels can maintain their efficiency with little resources or human intervention. The efficiency of solar panels, often built on arid landscapes, can be reduced by up to 40% as dust accumulates on the panels. Di Sun and Karl F. Böhringer, from the United States’ University of Washington, created a micro-patterned solar panel glass that incorporates hydrophilic, curved ‘rungs’ on a hydrophobic surface. When mechanical vibration is applied to the panel surface, water droplets are directionally conveyed along tracks of rungs at speeds of up to 27 mm per second, collecting and removing surface contaminants as they go. The system is simply and inexpensively fabricated and uses minimal amounts of water. In one test, a solar module achieved a 23% gain in power output after cleaning with the system.

## Introduction

Solar energy systems, including photovoltaic (PV) systems, concentrated photovoltaic (CPV) systems, and concentrated solar power (CSP) systems, are mostly built in semiarid or desert areas, where sun irradiance is an abundant resource but high levels of sand and dust particles are also present^1^. The accumulation of dust and environmental contaminants over time, which is often termed “soiling” or “fouling”, has become a growing concern for PV module efficiency and reliability^2^. Dust particles primarily consist of quartz and silicate minerals but also have lower concentrations of elements such as area-specific minerals, agricultural components, and fuel components. The general dust particle size distribution is in the 30~160 μm range^3,4^. Dust particles will accumulate on the cover glass of the solar panel modules and reduce the amount of light that reaches the solar cells to be converted to electricity. According to solar module monitoring results from the Thar Desert, India^1^, the conversion efficiency loss can be up to 40% over time. The cover glass is the first interface for the PV modules to interact with the incident photons. It is important to keep this interface clean to ensure the maximum solar power conversion efficiency. Mitigating the soiling of the PV module surfaces on a periodic basis is usually required to maintain efficiency. However, solar module installations are often inconvenient to access by maintenance staff. The cleaning of solar panel surfaces becomes problematic without labor-free and water-saving approaches.

Engineers have been exploring surface self-cleaning methods other than traditional cleaning to mitigate surface soiling and improve PV module efficiency. Learning from nature, researchers have adopted a “biomimetic” approach to create surface coatings replicating the micro-/nanomorphologies from lotus plant leaves^5–7^, rice leaves, butterfly wings^8–10^, and springtail cuticles^11,12^. On such surfaces with micro-/nanoroughness, water droplets can maintain high contact angles (CA > 150°), low sliding angles (SA < 10°) and low contact angle hysteresis (CAH < 10°). The adhesion force between the dust particles and the surface is reduced due to the reduced contact area. When the surface is tilted, the droplet will roll off the surface and remove the dust particles. The droplet is moved passively by gravity, and the cleaning path cannot be precisely defined to cover the entire surface area.

In addition to the passive self-cleaning surface approach, the droplet can be manipulated actively to dislodge surface contaminants by using other physical effects, such as electrical fields^13–16^, mechanical vibrational fields^17,18^, magnetic fields^19^, and acoustic wave fields^20–23^. In this paper, we designed and fabricated an active self-cleaning surface system by using a single droplet to systematically clean the surface contaminants. The system utilized patterned coatings and mechanical vibration. We created microsized hydrophilic curved rung structures on a hydrophobic background, which are termed anisotropic ratchet conveyors (ARCs). A droplet is first applied on the ARC patterned surface and then transported along a predefined ARC track that covers the entire surface area under mechanical orthogonal vibration. We discuss the droplet transport mechanisms by characterizing the anisotropic force, droplet transport speed, dependency on the vibrational frequency, and power consumption. The optical transmittance and solar power output performance are investigated on the assembled solar module level. A proof-of-concept cleaning system was demonstrated by assembling an ARC-coated soda-lime cover glass on solar cells.

## Results

### ARC system design and material property characterization

**Fig. 1:**
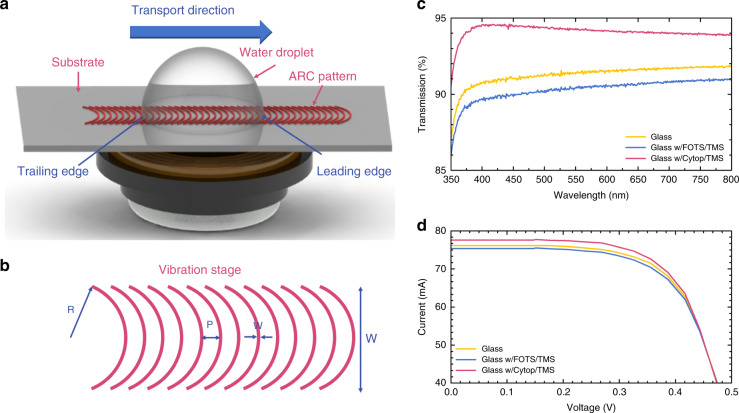
System design and optical performance characterization. **a** Self-cleaning surface system design. **b** Top view of the ARC design. The parameters include the rung radius of curvature (*R*), ARC rung center-to-center period distance (*P*), hydrophilic region width (*w*), and the total ARC track width (*W*). **c** Optical transmittance measurement results with a wavelength range of 350~800 nm. Ten-centimeter soda-lime glass wafers were used as the substrate baseline. **d** I-V output of solar module assembly with a coated cover glass.

A design schematic of our ARC self-cleaning system is shown in Fig. 1a. Hydrophilic curved rungs are patterned on the hydrophobic background on the substrate (silicon wafer or soda-lime glass). Different material combinations with hydrophobic/hydrophilic behaviors can be applied. In our work, we used a perfluoro-octyltrichlorosilane (FOTS)–trimethylsilanol (TMS) self-assembled monolayer (SAM) system and a Cytop–TMS spin-coated thin-film system. Table 1 summarizes the material properties, including static CA, dynamic CA hysteresis, sliding CA on an inclined surface and coating thickness. The SAM is created by chemisorption of the trichlorosilane “headgroups” to the hydroxyl group on the substrate, forming a stable covalent bond. The functional “tail group” can be altered, providing different surface energies to create hydrophobic/hydrophilic contrast. The FOTS “tail group” is highly fluorinated; thus, the surface energy is reduced after treatment to provide a hydrophobic surface finish. The deposition of the SAM can be either in the vapor phase or in solution. The SAM coating is only molecular-level thick, making the coating transparent and optically flat.

**Table. 1:** Characterization results of surface properties for different coating materials.

Material	CYTOP	FOTS	TMS
Static CA (°)	110	108	75
Advancing CA (°)	115	117	79
Receding CA (°)	101	88	64
Hysteresis (°)	14	29	15
Sliding angle (°)	22	77	22
Thickness (nm)	70	Molecular monolayer	Molecular monolayer

Sliding angles were measured with a 15 μL water droplet on a polished silicon wafer substrate. Cytop thickness was measured with a stylus profilometer.

Cytop is an amorphous fluoropolymer with good transparency over the visible and UV wavelength ranges, good solubility to coat various surface designs, and excellent water repellent properties. The refractive index of Cytop is 1.34, which allows the material to serve as an anti-reflective coating on glass substrates. Micropatterning on hydrophobic surfaces (such as Teflon and Cytop) is difficult using standard photolithography due to poor adhesion between the photoresist and Cytop. Methods have been proposed using a metal buffer layer^24,25^ or plasma pretreatment of the surface, but the original hydrophobic surface properties will be damaged after treatment with decreasing water droplet CA.

Poly(*p*-xylylene) polymers are a special series of polymers that produce uniform pinhole-free films in a chemical vapor deposition process. Parylene can be etched with oxygen plasma, making it compatible with standard lithography processes. It has been used to create biomolecular stencil arrays^26^ and patterns on soft substrates^27^. By adopting parylene-C as a stencil mask, we created hydrophilic patterns on top of the Cytop surface without degrading its original surface properties. The characterization results of the surface properties of the coating materials used in this paper are shown in Table 1. SEM images of the patterned Cytop are shown in Supplementary S1.

### Optical transmittance and solar module output

It is important to understand the optical performance of the ARC patterned coating. We performed optical transmission measurements, as shown in Fig. 1c,d. For the FOTS-TMS system, the light transmission was degraded due to the added coating of monolayers, but within a range of less than 1%. The glass after FOTS-TMS treatment was transparent and optically flat. Furthermore, the Cytop-TMS system improved the transmission with an enhancement of 2.5%~3.5% over the visible wavelength range even with an added coating on top of the glass. The reason was that the refractive index of Cytop is ~1.34, which is between those of air (*n*_air_ = 1.0) and the glass substrate (*n*_glass_ = 1.5), providing a refractive index match. Similar to Rayleigh’s film, a portion of the incoming light reflects both at the interface of air/Cytop and Cytop/glass but has less reflection than the single reflection at the air/glass interface with a larger refractive index mismatch. Figure 1d shows the I-V curve measurements for assembled PV modules. The ARC structure was patterned over a 5 cm by 5 cm solar cell surface. The Cytop-TMS coating generated higher optical output power than bare glass and FOTS-TMS surface treatment, in accordance with the light transmission measurements. The optical performance demonstrated that our coating systems were compatible with solar module cover glass and can even have anti-reflective properties to improve solar module power output efficiency.

### Droplet transport characterization

**Fig. 2:**
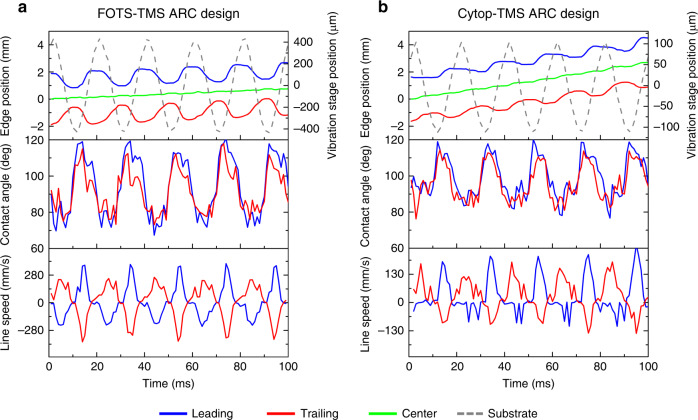
Droplet leading and trailing edge position, contact angle, and line spreading speed with time. We define the line speed as the derivative of the droplet edge position over time. **a** FOTS-TMS ARC design. The vibration stage acceleration and peak amplitude are 4.1 g and 0.41 mm, respectively, at 50 Hz. **b** Cytop-TMS ARC design. The vibration stage acceleration and peak amplitude are 1.1 g and 0.11 mm, respectively, at 50 Hz. *g* = 9.8 m/s^2^.

The test wafer with ARC patterns was mounted on a vibration stage. A 10 μL (2.84 mm in diameter) water droplet was pipetted on the surface, and the droplet silhouette was monitored via a high-speed camera with a frame rate of 1000 fps. We tested both FOTS-TMS and Cytop-TMS systems, with the design parameters of *R* = 1000 μm, *P* = 100 μm, *w* = 10 μm, and *W* = 1.8 mm. Figure 2a, b shows a typical droplet leading and trailing position change, CA change and line speed with time as the substrate vibrated orthogonally. The droplet transport speed was 7.5 mm/s on the FOTS-TMS ARC surface and 27 mm/s on the Cytop-TMS ARC surface. In this design, we translated substrate orthogonal vibration into droplet lateral expansion and recession and thus moved the droplet by the anisotropic forces at the leading and trailing edge where the solid–liquid–gas three-phase contact line resided. A detailed theoretical derivation of the anisotropic force can be found in reference^28^. With the aid of the ARC, the droplet can overcome the force of gravity and climb on inclined surfaces under orthogonal vibration. Our experiment showed that the droplet can climb uphill at up to a 15° inclination of the surface. With higher inclination angles, the droplet tended to be “shaken off ” the surface.

### The frequency response of the droplet transport

**Fig. 3:**
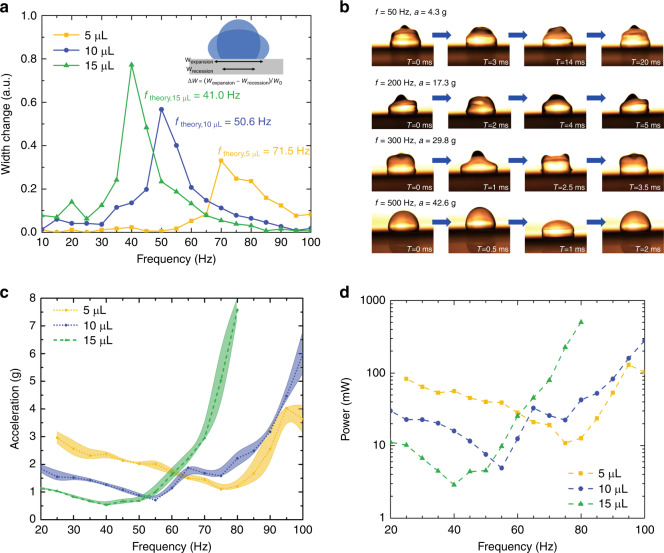
Self-cleaning surface system frequency response characterization. **a** Frequency response of water droplets with different volumes on Cytop surfaces under the same vibration acceleration amplitude of 1 g. The initial droplet width *w*_0_ on the Cytop surface was measured as 2.24, 2.87, and 3.39 mm separately by image processing. **b** Water droplet (7 µL) transport modes under different frequencies. The ARC track requires vibration input with higher accelerations to drive the water droplet forward as the frequency increases. **c** Minimum vibration acceleration map required to transport the droplet. Three droplets were measured at each frequency point. Data are shown ±one standard deviation for *n* = 3. **d** Estimated power consumption of the electromagnetic shaker. The overall load weight was measured at 200 g. The spring constant of the system is 12 N/mm according to the instrument datasheet.

It is important to understand the frequency response of the droplet and to match it with the dynamic behavior of the PV modules^29^. The water droplet exhibits different resonance modes depending on mass and surface tension. The *n*^th^ resonance mode of the droplet can be expressed as^30–32^

1 $$f_n = \frac{\pi }{2}\left( {\frac{{n^3\gamma }}{{24m}}\frac{{\mathrm{cos}^3\theta - 3\cos \theta + 2}}{{\theta ^3}}} \right)^{\frac{1}{2}}$$

where *n* = 2, 3, 4,… is the mode number, *γ* is the water surface tension (in N/m), *θ* is the CA (in radians) and *m* is the water mass (in kg). We modeled the droplet on the uniform hydrophobic surface under vibration as a forced mass-spring oscillator system^33^ and then characterized the water drop resonance frequency in low-frequency bandwidth regions (10–100 Hz, at every 5 Hz) by monitoring the droplet width change at different vibration frequencies. The vibration acceleration amplitude was kept constant at 1 g, meaning the droplet was driven by a periodic external force. Figure 3a shows the plot of the relative droplet width change of 5, 10, and 15 μL droplets at different frequencies. Due to the mechanical resonance behavior of the droplet, it was relatively easy to drive the center volume of the droplet on the substrate using frequencies close to its resonance when relatively low orthogonal vibration energy was required to achieve enough droplet sideway expansion amplitude. Figure 3b shows examples of droplet transport with higher modes on ARC surfaces at 50, 200, 300, and 500 Hz. The acceleration required to move the water droplet along the ARC rises as the mode number increases.

To study the required power input to drive the droplet, if we ignore the mass of the substrate, we have the following expression describing a damped oscillator with a harmonic driving force^34^

2 $$\frac{{\mathrm{d}^2}}{{\mathrm{d}t^2}}x\left( t \right) + {\mathrm{\Gamma }}\frac{\mathrm{d}}{{\mathrm{d}t}}x\left( t \right) + \omega _0^2x\left( t \right) = \frac{{F_0\cos \left( {\omega _dt} \right)}}{m}$$

where *x*(*t*) is droplet position with time, Γ is the damping constant of the electromagnetic vibration exciter (in s^−1^), *F*_0_ is the driven force, *ω*_0_/2*π* is the natural frequency of the oscillator and *ω*_*d*_/2*π* is the driven frequency. The solution to the equation above is

3 $$x\left( t \right) = A\cos \left( {\omega _dt} \right) + B\sin (\omega _dt)$$

4 $$A = \frac{{\left( {\omega _0^2 - \omega _d^2} \right)a}}{{(\omega _0^2 - \omega _d^2)^2 + {\mathrm{\Gamma }}^2\omega _d^2}}$$

5 $$B = \frac{{{\mathrm{\Gamma }}\omega _da}}{{(\omega _0^2 - \omega _d^2)^2 + {\mathrm{\Gamma }}^2\omega _d^2}}$$

where *A* is the elastic amplitude and *B* is the absorptive amplitude. $a = \frac{{F_0}}{m}$ can be measured from experiments. The average power within any single oscillation period is related to the *B* term

6 $$P = \frac{1}{T}\mathop {\int }\limits_{0}^{T} F\frac{{\mathrm{d}{\it{x}}}}{{\mathrm{d}t}}\mathrm{d}t = \frac{1}{2}{\it{F}}_{0}\omega_{\it{d}}{\it{B}} = \frac{1}{2}{\it{m}}a\omega_{\it{d}}{\it{B}}$$

To estimate the power required to drive the droplet, we measured the minimum acceleration required to drive the droplet over the 20~100 Hz frequency bandwidth, as shown in Fig. 3c. We then calculated the average power based on Eq. (6). All the parameters could be inserted through measurement or datasheet, except for the damping coefficient of the electromagnetic vibration exciter. We estimated a large damping coefficient Γ = 1000 s^−1^ in our calculation based on the fact that the electromagnetic vibration exciter was a highly damped system and the conversion efficiency from electrical to mechanical was low at its maximum load (~0.1%) among the working frequencies^35^. The power calculations based on experimentally measured data on Cytop-TMS ARC designs are presented in the Discussion section below.

### Surface anisotropic force by SA measurement

**Fig. 4:**
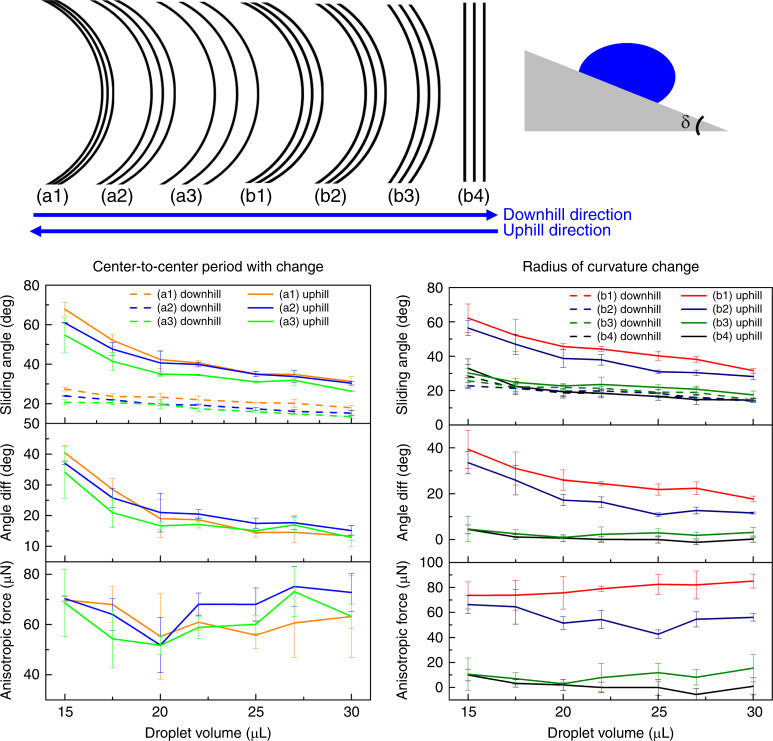
Anisotropic force measurements with the sliding test. **a** Sliding measurement results on different ARC designs. The radius of curvature of the ARC pointed downhill or uphill. The critical sliding angle was recorded from the accelerometer when the droplet started to slide off the surface. The center-to-center period widths (*P*) for groups (**a1**), (**a2**), and (**a3**) were 50, 100, and 200 µm, respectively, and the other parameters were *R* = 1000 µm, *w* = 10 µm, and *W* = 1800 µm. The radii of curvature (*R*) for groups (**b1**), (**b2**), (**b3**), and (**b4**) were 1000, 1100, 1500 µm, infinity (straight lines), and other parameters were *P* = 100 µm, *w* = 10 µm, and *W* = 2000 µm. Three droplets were used for each measurement. Data are shown ±one standard deviation for *n* = 3.

To evaluate the anisotropic forces, a slip test was performed with different ARC designs (Fig. 4). The inclination angle is defined when the droplet starts to slide off the tilted surfaces. The gravity of the water droplet overcomes the surface adhesion at the inclination angle. The ARC radius of curvature is pointed either uphill or downhill on inclined surfaces, as indicated in Fig. 4. The anisotropic pinning force can be expressed as

7 $$F_{\mathrm{anis}} = F_{\mathrm{slip,uphill}} - F_{\mathrm{slip,downhill}} = mg\sin \delta _{\mathrm{uphill}} - mg\sin \delta _{\mathrm{downhill}}$$

where *δ* is the surface inclination angle. We performed a slip test on the Cytop-TMS ARC surface. For the (a1)–(a3) design group, we changed the center-to-center period width (*P*) to 50, 100, and 200 μm while keeping the other parameters the same. For the (b1)–(b4) design group, we altered the ARC pattern radius of curvature (*R*) with 1000, 1100, 1500 μm, and straight lines. As seen from the results of the change in period (a1)–(a3), the SA for both uphill and downhill decreases as the period gap increases. The anisotropic forces remained similar for different designs. However, we could observe a decrease in the anisotropic force as we increased the ARC radius of curvature from the measurement results of (b1)–(b4). The average anisotropic force on a radius of curvature *R* = 1000, 1100, and 1500 μm was 73, 66, and 11 μN, respectively. When there were only straight hydrophilic lines, no SA differences were observed for uphill and downhill measurements, and the anisotropic forces were close to zero. To achieve a better ratcheting performance, we chose the ARC radius of curvature *R* = 1000 μm to drive the droplet to move.

## Discussion

### Dust particle adhesion force and water droplet cleaning force

Several parameters influence the surface interactions between dust particles and the surfaces they deposit on, including particle material characteristics, environmental conditions (relative humidity and temperature), surface roughness and treatments^36^. Theoretical conceptualization of the particle mobilization caused by the advancing and receding of the three-phase contact line (TPL) is based on the analysis of the major forces acting on the dust particles. The forces mainly include adhesion forces and detachment forces (surface tension force and hydrodynamic force). For small dust particles (diameter < 500 µm), the adhesion forces are mainly van der Waals forces and the electrostatic force in dry environments, as well as the capillary force in humid environments^3,37^. The adhesion force for a single particle on a surface can be in the range of 100~300 nN, as characterized by an AFM tip^3^.

As a droplet is applied on the contaminated surface and transported under vibration, dust particles will be removed from the surface by the droplet, demonstrating the dominance of detachment forces over adhesion forces. There are two main sources of detachment force: capillary force and hydrodynamic shear force.

**Fig. 5:**
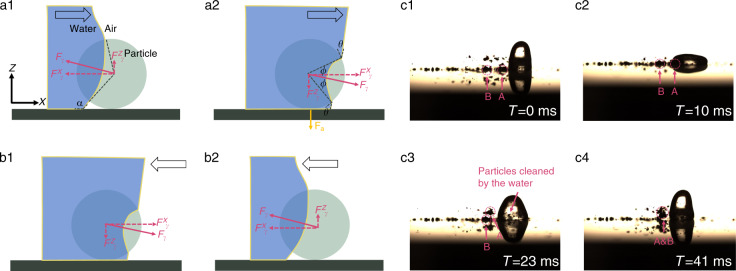
Surface cleaning force model and cleaning process demonstration. (**a**1) and (**a**2) show the direction of the surface tension force when the TPL is advancing to immerse the dust particles on the surface. (**b**1) and (**b**2) show the direction of the surface tension force when the TPL is receding away from the dust particles on the surface. (**c**1)–(**c**4) demonstrate how the bulk hydrophilic SiN_x_ powder is detached from the surface and relocated by the interfacial surface tension. When the droplet expands, the water-air interfacial force removes powder particles from point A to point B. Eventually, these particles will be trapped inside the droplet and carried away with the moving droplet.

When a water-air interface comes in contact with dust particles, as shown in Fig. 5, the capillary force at the interface can be expressed as^38,39^

8 $$F_\gamma = 2\pi R\gamma \sin \phi \sin \left( {\theta - \phi } \right)$$

where *γ* is the water/air surface tension (72 mN/m at 25 °C), *R* is the radius of the spherical dust particle, *θ* is the CA between the water and the dust particle, and *ϕ* is the filling angle describing the position of the water/air interface on the particle surfaces. We have the maximum capillary force on the vertical and horizontal directions^36^ when $\phi = \frac{\theta }{2}$

9 $$F_{\gamma ,{\mathrm{max}}}^z = 2\pi R\gamma \sin ^2\frac{\phi }{2}\cos \alpha$$

10 $$F_{\gamma ,{\mathrm{max}}}^x = 2\pi R\gamma \sin ^2\frac{\phi }{2}\sin \alpha$$

where *α* is the CA between water and the substrate. Since the particle sizes are small, the buoyancy force and the gravity force are negligible. As the droplet is being actively agitated by vibration, the dust particles are removed by the advancing and receding of the water-air interfaces. The dust particles will be brought inside the water droplet due to the internal circulating flow of the vibrating droplet^40^.

In addition, the moving water/air interface exerts a hydrodynamic shear force on the particles. If the shape of the particle is spherical, then the maximum tangential shear force, which occurs when the particle is completely exposed to the moving fluid, can be expressed as^41^

11 $$F_s = 1.7(6\pi )\eta Rv$$

where *F*_*s*_ is the shear force on the particle, *η* is the water dynamic viscosity (8.9 × 10^−4^ Pa s) and *v* is the fluid water/air spreading speed.

To estimate the cleaning capillary and shear force, we assumed a droplet line speed on the order of 200 mm/s based on previous characterization results. The radius of the nitride particle was 200 μm, with a water/nitride CA of 73° and a water/Cytop CA of 110°. Based on the theoretical calculation provided by Eqs. (9) and (10), the surface tension forces were on the order of 10 μN in the vertical direction and 30 μN in the horizontal direction. The maximum shear force from the droplet leading-edge expansion is on the order of 1 μN from Eq. (11). Compared with the adhesion force measurement results, the surface tension force and water hydrodynamic shear force are sufficient to remove the surface contaminants as the droplet moves onto the surface contaminants. Figure 5c1~c4 demonstrates the cleaning process for particles (SiN_*x*_ powder) by a moving water droplet under the high-speed camera. Dust particles were dislodged by the surface tension force and the hydrodynamic force while encapsulated by the internal flow of the vibrating droplet. The droplet was able to carry the SiN_*x*_ particles along. Similar phenomena could also be observed with carbon powder.

### Self-cleaning surface design: FOTS-TMS ARC zig-zag pattern

**Fig. 6:**
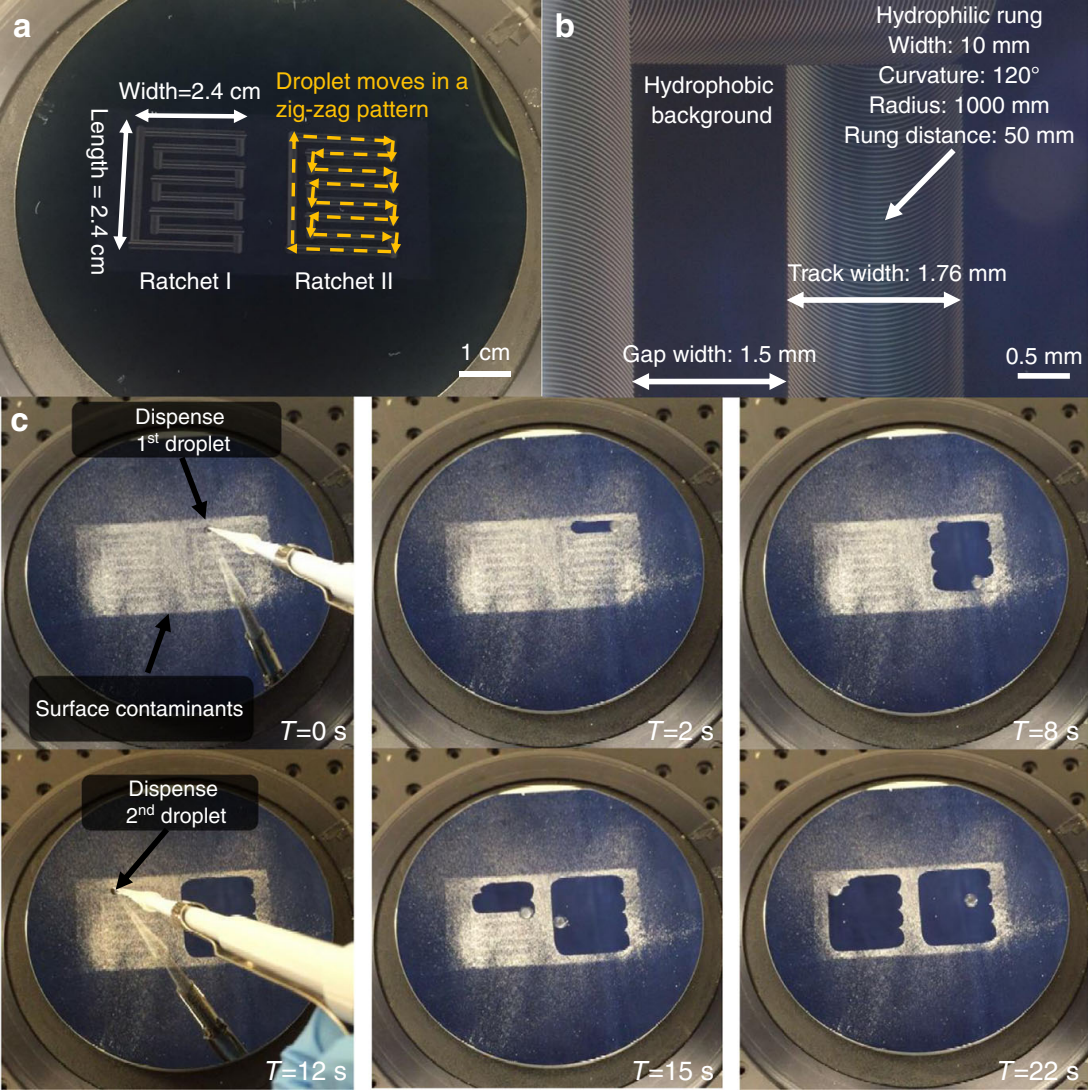
Self-cleaning surface demonstration on the Si substrate. **a** Entire ARC track design after lithographic patterning. The droplet follows the zig-zag pattern on the ARC tracks. The photoresist pattern is removed after FOTS deposition, leaving only a transparent and optically flat monolayer surface. **b** ARC track pattern at the corner after lithographic patterning. **c** Surface cleaning performance for sweetener (dextrose, maltodextrin, and sucralose) contamination on the ARC surface consisting of two ARC tracks shown in (**b**). The demo video is shown in Supplementary Movie S1.

To create a surface cleaning system with a droplet, we designed a zig-zag pattern of the ARC, as shown in Fig. 6. The droplet will travel along the defined zig-zag pattern, looping around the cleaning area. We used a 10 μL droplet on an ARC track with a width of 1.75 mm and an edge-to-edge gap distance of 1.5 mm. The droplet footprint had overlapping areas while moving on the adjacent tracks to fully cover the surface. The total surface area is approximately 5.76 cm^2^ (2.4 cm by 2.4 cm). At the corners, the droplet must abruptly change its velocity to the perpendicular moving direction. We iterated our designs and selected the design shown in Fig. 6b by adjusting the relative position of the horizontal and perpendicular tracks at the turning corner. Figure 6c represents subsequent video frames of the cleaning process of sweetener particles on the surfaces. The water droplet was applied on the first loop at time *T* = 0 s and started the cleaning process. The vibration frequency was 50 Hz. At *T* = 12 s, a second droplet was applied on the second loop surface. All sweetener particles on the ARC areas were cleaned by the droplet. We observed a slowing motion of the droplet as it collected more sweetener, which increased its mass and viscosity.

### Self-cleaning surface design: Cytop-TMS ARC zig-zag pattern design

**Fig. 7:**
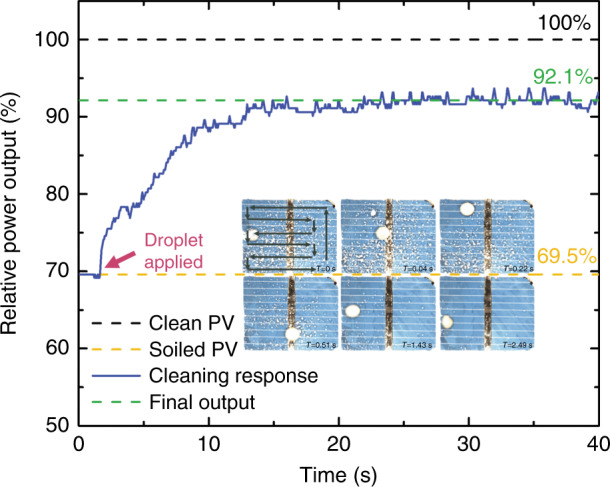
Real-time power output response of a 30 mm by 24 mm polysilicon solar module during the cleaning process. The module was connected to a resistive load for power monitoring. The insert shows the top view of the cleaning process with three continuous cleaning cycles. The droplet is 13.5 µL in volume at a vibration frequency of 50 Hz. The droplet zig-zag path is shown at *T* = 0 s with the black dotted line. The demo video is shown in Supplementary Movie S2.

We then created a Cytop-TMS ARC on the soda-lime cover glass of an in-house assembled PV module. An a-Si solar cell was cut to 30 mm by 24 mm and glued between the cover glass and an acrylic substrate. Silicon nitride powder was applied (with a total mass of 10 mg) on the surface through a polyester mesh with a pore size of 250 μm. A 13.5 μL droplet was pipetted on top and followed the zig-zag ARC track while cleaning the surface contaminants. The response of the solar module output efficiency was monitored in real-time with Arduino and MATLAB. As the droplet picked up nitride particles, the solar output efficiency started to increase. For one measurement shown in Fig. 7, we observed a 23 percentage-point gain by cleaning, from 69.1% after contamination to 92.1% after 15 s of cleaning. We observed particles bouncing on the solar cell surfaces from time to time that were captured as the droplet swept by. Due to the irregular motion of the dust particles bouncing on the vibrating surface, a longer cleaning process was anticipated to clean all the dust particles on top of the solar cell. We estimate that more than 80% of the applied SiN_*x*_ powder was collected by the droplet during the first 20 s of the recording period. The remaining particles might have bounced out of the cleaning area due to the external mechanical vibration. A single 13.5 μL water droplet could carry at least 8 mg of particles and move them along the ARC track. Depending on the application requirements, different ARC track designs can be implemented to guide the droplet movement path. The dirty droplets, after collecting the surface contaminants, can be directed off the edge of the cover glass or collected at the end of an ARC track without a looping path. The collection area should avoid the solar cell surface underneath.

The total energy consumption of the vibration system is proportional to the cleaning time. In our case, as shown in Fig. 7, the droplet cleaning time for a 30 mm by 24 mm area (720 mm^2^) was *T*_clean_ = 15 s, compared to an average sunshine time of *T*_sun_ = 8 h for power generation by the solar modules during a single day. The polysilicon solar cell we used has an average power rating of ~0.16 mW/mm^2^. We define *P*_1_ as the power consumption for cleaning the system shown in Fig. 7 (*P*_*1*_ ≈ 7 mW, estimated from Fig. 3d), *P*_2_ as the power generation for a clean solar module (*P*_*2*_ ≈ 0.147 mW/mm^2^) and *P*_3_ as the power generation per surface area for a soiled solar module (*P*_*3*_ ≈ 0.111 mW/mm^2^). Then, we can calculate the energy gained due to cleaning as $\left( {P_2 - P_3} \right) \cdot T_{{\mathrm{sun}}} \cdot 720\;{\mathrm{mm}}^2 \approx 207\;{\mathrm{mWh}}$, while the energy consumption due to cleaning is $P_1 \cdot T_{{\mathrm{clean}}} \approx 0.03\;{\mathrm{mWh}}$. Even when considering an efficiency of only 0.1% for the conversion from electrical to mechanical power, the vibration system would consume only 30 mWh. This result indicates that we can substantially improve the overall daily solar output energy by keeping the surface clean with our self-cleaning system. To further reduce the system power consumption, a better vibration system should be designed to reach the minimum power consumption at the resonance frequency of the surface cleaning droplet.

We tested the surface cleaning function with different categories of typical contamination, including dust and dirt particles and soluble and insoluble contaminations, summarized in Supplementary S2. Most water-soluble materials (such as salt and sweetener) and low surface adhesion insoluble particles (such as sand, SiO_*x*_, SiN_*x*_, carbon powers) can be effectively cleaned with the droplet from the self-cleaning surface. Hydrophobic surface contaminants such as polytetrafluoroethylene (PTFE) tended to stay at the boundary of the water droplet instead of being trapped inside the droplet. The droplet surface was gradually covered with solid powder so that the surface tension at the TPL was reduced. Eventually, a “droplet marble” was formed that jumped on the surface when agitated by vibration.

## Conclusion

In this paper, we demonstrated a proof-of-concept self-cleaning surface system with ARC tracks using both FOTS-TMS and Cytop-TMS coatings. Cytop was successfully patterned using parylene as the stencil mask. Solar module optical transmission and solar cell power I-V measurements were performed, demonstrating that the Cytop coating can provide antireflection properties on soda-lime cover glass and improve solar output efficiency. Different surface contaminants, including water-soluble, insoluble, hydrophobic and hydrophilic dust particles, were characterized. Our system relies on the surface tension anisotropy at the TPL of the droplet boundary to move the droplet while dislodging and removing surface contaminants during droplet expansion and recession phases under vibration. Compared with hydrophobic surfaces based on the lotus effect, our system has the advantage of systematically transporting the droplet to designated locations compared with uncontrolled droplet rolling by gravity on hydrophobic surfaces. The cleaning process only utilizes a sessile droplet with minimized water usage. The entire fabrication consists of a single-mask process to reduce manufacturing costs.

## Materials and methods

### Device fabrication

#### FOTS-TMS self-assembled monolayer system

The fabrication process flow for the FOTS-TMS system is shown in Supplementary S3. A silicon wafer was first cleaned with a piranha solution at 110 °C for 10 min, rinsed with deionized (DI) water and dried with nitrogen gas using a spin rinse dryer (ClassOne Technology, Inc.). The wafer was then put into the priming oven and treated with hexamethyldisilazane (HMDS) vapor at 150 °C, forming a TMS SAM on the silicon oxide. A 1.2 μm photoresist AZ1512 was spin coated on the wafer surface, and the ARC pattern was directly exposed with a Heidelberg-MicroPG-101 mask writer (Heidelberg Instruments Mikrotechnik GmbH). The wafer was developed with an AZ340 photoresist developer (AZ340: DI water = 4:1) and treated with oxygen plasma for 1 min at 50 W in a barrel asher (Glow Research) to etch away the exposed TMS layer. The wafer was immersed in FOTS vapor inside the vacuum chamber for 1 h. Then, the photoresist was washed away with acetone, isopropanol alcohol, and DI water before drying. Before testing, we annealed the wafer at 150 °C for 20 min. The fabrication is a one-mask process that minimizes the fabrication cost and turn-around time.

#### Cytop-TMS thin-film system

To fabricate the Cytop-TMS thin film surfaces (shown in Supplementary S3), a silicon wafer was first cleaned with piranha solution at 110 °C for 10 min, rinsed with deionized (DI) water and dried with nitrogen gas using a spin rinse dryer (ClassOne Technology). After Si/glass wafer cleaning, diluted Cytop (Cytop CTL-809M: CTL-Solv.180 = 3:1) was spin-coated on a silicon wafer and baked at 110 °C for 20 min followed by 1-h baking at 200 °C. Then, 2.5 μm parylene was evaporated on the Cytop using a commercial parylene coater (PDS 2010, Specialty Coating Systems) under vacuum. A 6 μm photoresist (AZ9620) was coated and patterned with the Heidelberg mask writer. The parylene stencil mask and Cytop were etched through O_2_ plasma using reactive ion etching (Vision RIE). Then, the parylene stencil mask was peeled off with tweezers. The surface was treated with spin-on TMS (MicroPrime MP-P20) and baked at 110 °C for 2 min.

### Vibration stage setup

The testbench setup is shown in Supplementary S4. The test wafer was mounted on an aluminum platform, which is attached to the vibration exciter (Brüel and Kjær type 4809) with double-sided adhesive tape. A sinusoidal wave signal was generated by the function generator and amplified by the power amplifier to drive the vibration exciter (Brüel and Kjær type 2718). The vibration amplitude was monitored with a vibrometer (Polytec OFV) with an oscilloscope (Agilent Infiniium). The water droplet movement was captured by a high-speed camera (FASTCAM Mini UX100) at 1000 fps. The image was processed with MATLAB custom code to generate the position and CA change of the droplet leading and trailing edges with time. The conversion between the droplet width and pixel size was calibrated with a standard calibration bead with a diameter of 3 mm.

### Light transmittance and solar power monitoring

Light transmission data of the two ARC surface coatings on a soda-lime glass substrate were obtained with a Cary 5000 UV-Vis-NIR spectrophotometer (Agilent Technologies, Inc.). A customized source meter was used for the I-V curve output of the solar modules, as shown in Supplementary S4. We used an Arduino Nano development board, which was based on the ATmega328P microcontroller. The board was powered by a Mini-B USB and had 14 digital pins as input or output. Eight analog pins were configured as analog voltage reading input, with 10-bit resolution. We used an MCP4822 dual-channel digital-to-analog (DAC) chip to create voltage bias on the solar modules. We configured TLV4110 high current operational amplifiers as voltage followers, in which the op-amp can handle up to 500 mA output current. A 1 Ω resistor was used as the current sensing resistor, and an AD8210 was used as the current monitoring chip. The entire system was designed to use a single power supply, so an AD780 high precision reference was configured at 2.5 V as the DC shift from ground. The Arduino took voltage output from the current sensing chip and converted the analog data to digital through the 10-bit resolution analog port.

### CA and SA measurement setup

Both the water droplet static CA and dynamic CA on uniform coated FOTS and Cytop surfaces were measured with a Krüss Drop Shape Analyzer (DSA 100). For dynamic CA measurements, the water dispensing and retreating speed was 10 μL/s with a total volume of 30 μL. For the droplet SA measurements, a custom experimental setup was designed, as shown in Supplementary S4. The test wafer was mounted on an acrylic tilting stage, and a 3-axis accelerometer (MMA8451 from Adafruit) was attached to the stage. The accelerometer output was communicated to an Arduino microcontroller through I2C communication pins. The inclination angle was derived from the accelerometer output of the *x*-, *y*-, and *z*-axes. The ARC track radius of curvature pointing either upward or downward was tested by applying a droplet on the measurement ARC track and observing the tilting angle while the droplet started to slide off the surface by gravity.

## Supplementary information

Supplementary Movie S1

Supplementary Movie S2

Supplementary Information
