# Supplementary material for: An active self-cleaning surface system for photovoltaic modules using anisotropic ratchet conveyors and mechanical vibration
Source: Microsyst Nanoeng. 2020 Sep 21;6:87. doi: 10.1038/s41378-020-00197-z (PMC8433153; doi:10.1038/s41378-020-00197-z)
Supplement: Supplementary file 3 — Supplementary Information [file 41378_2020_197_MOESM3_ESM.docx]

An Active Self-cleaning Surface System for Photovoltaic Modules Using Anisotropic Ratchet Conveyors and Mechanical Vibration

Di Sun^1,2^ & Karl F. Böhringer^1,2,*^

1. Electrical & Computer Engineering Department, University of Washington, Seattle, WA 98195, USA

2. Institute for Nano-Engineered Systems, University of Washington, Seattle, WA 98195, USA

**Corresponding Author:* Email: karlb@uw.edu; Tel: 206 221-5177; Fax: 206 543-3842

**S1 Patterned Cytop Surface**

The patterned Cytop after etching and peeling off the parylene shadow mask is shown in FIG. S1. The ARC patterns were successfully patterned on Cytop thin film with smooth edges. Cytop hydrophobicity was not degraded after peeling off parylene.


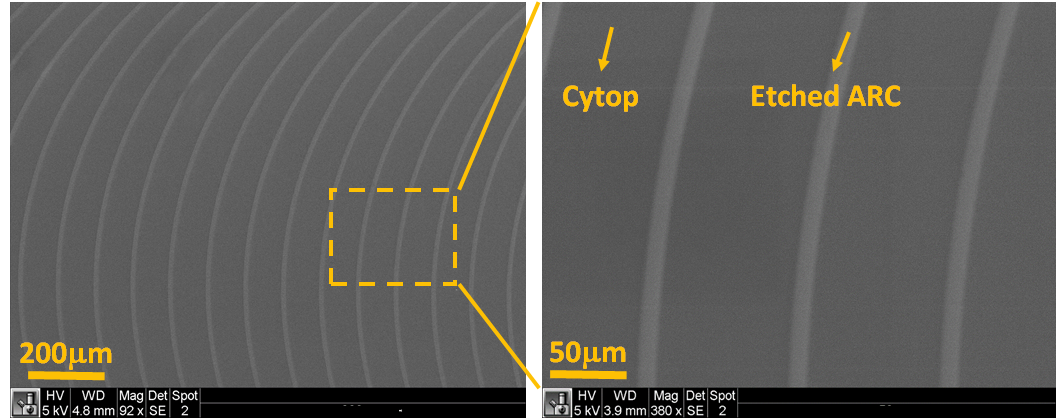


FIG. S1. SEM images of ARC patterns on Cytop. For each semi-circular rung, the radius of curvature (*R*) is 1000 μm, the etched linewidth (*w*) is 10 μm, and the period (*P*) between adjacent rung centers is 100 μm.

**S2 Dust particle types tested with self-cleaning surface systems**

Table: Materials tested on the self-cleaning surface.

| **Material** | Sand  (< 600 µm) | Salt* | Sweetener** | SiO_x_ and SiN_x_ | Carbon powder | PTFE |
| --- | --- | --- | --- | --- | --- | --- |
| **Can be cleaned or not** | Y | Y | Y | Y | Y | N |

*The salt is sodium chloride.

** The major contents of the sweetener are dextrose, maltodextrin, and sucralose.

**S3 Process Flow Diagram**

The detailed process flow diagram is shown in FIG. S3 below:


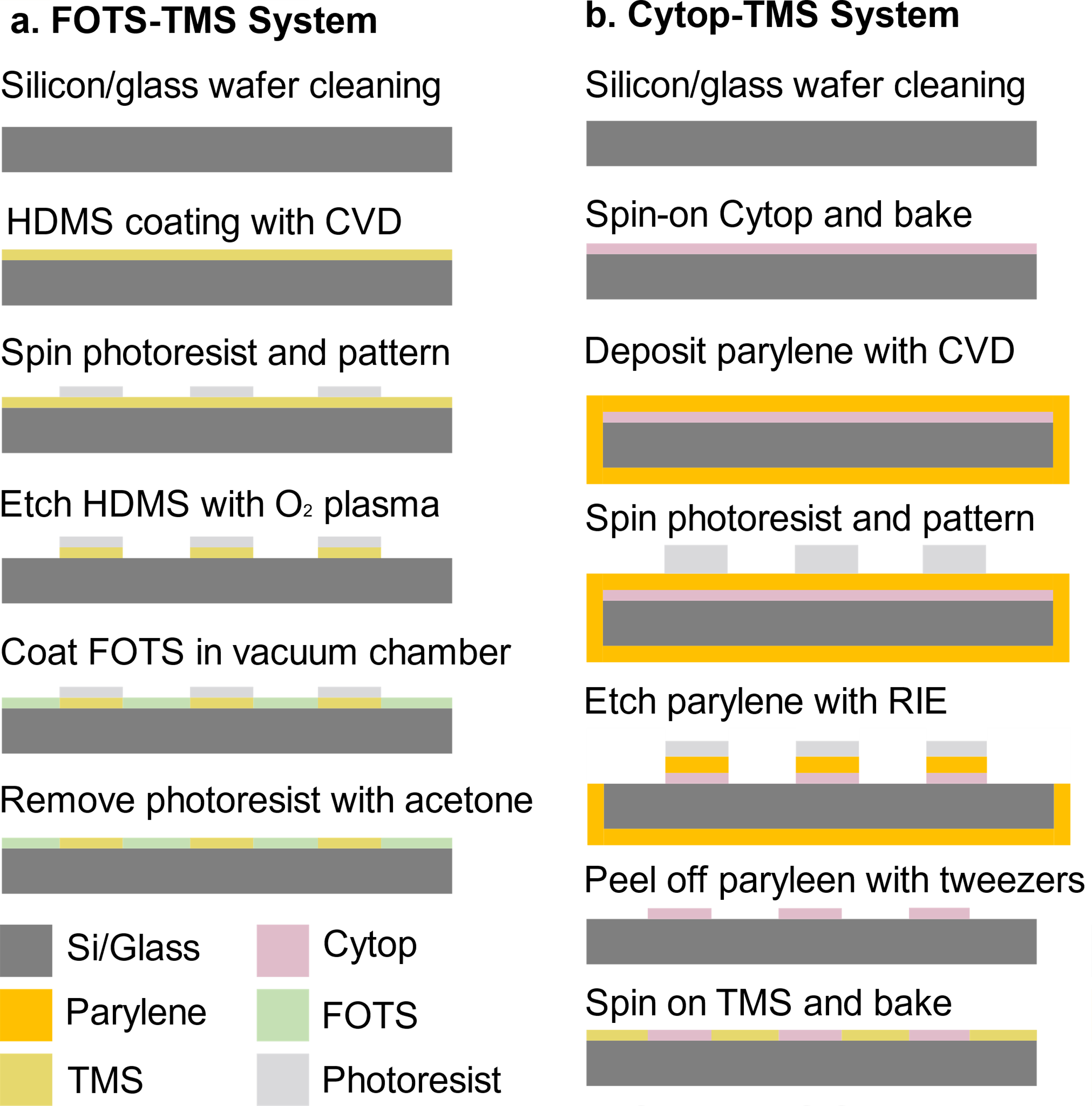


FIG. S3. (a) The fabrication process of the FOTS-TMS system. (b) The fabrication process of the Cytop-TMS system.

**S4 Experimental test bench setup**

Diagrams and pictures of the characterization test bench are shown in FIG. S4 below:


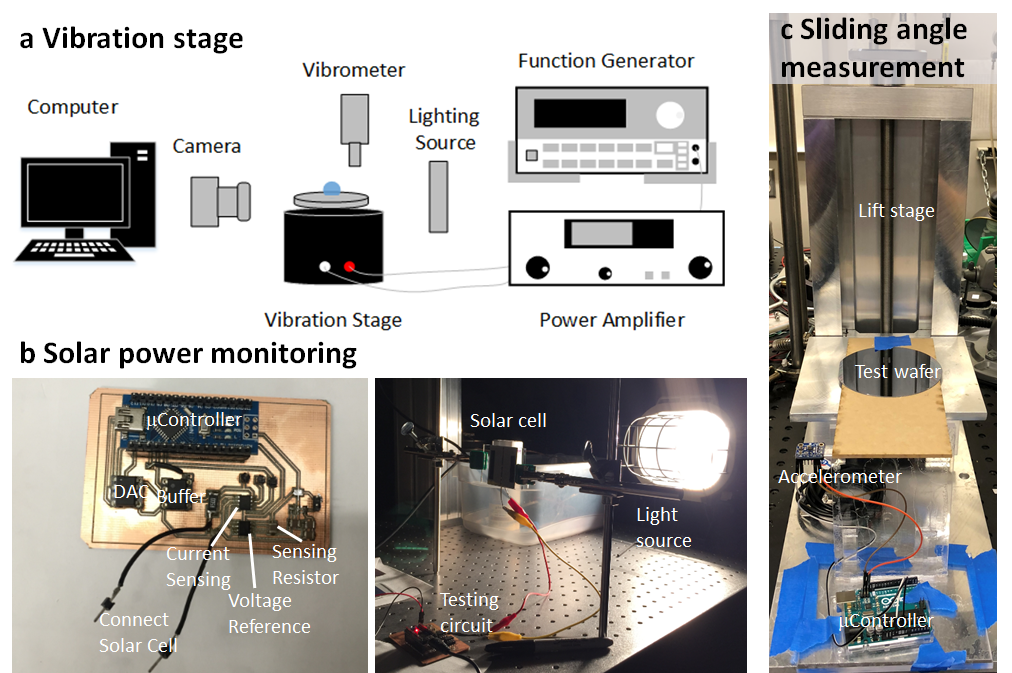


FIG. S4. (a) Diagram of the vibration stage experimental setup. (b) PCB layout of the source meter design and I-V curve measurement testing setup. (c) Custom-made inclination angle measurement setup.

Movie S1. Self-cleaning surface with zig-zag ARC tracks

Movie S2. Real time solar module cleaning
